# Design and Experimental Validation of a Flexible-Hinge-Based Manual Mechanism for Micro/Nano-Displacement Scaling

**DOI:** 10.3390/mi17030323

**Published:** 2026-03-05

**Authors:** Songling Tian, Meirun Gao, Yiyi Fu, Chenkai Fang, Xiaofan Deng, Liangyu Cui

**Affiliations:** 1 School of Control and Mechanical Engineering, Tianjin Chengjian University, Tianjin 300384, China; tiansl201911@tcu.edu.cn (S.T.); 18835418622@163.com (M.G.); 2 School of Mechanical Engineering, Tianjin University of Technology and Education, Tianjin 300222, China; fyy3223373112@163.com (Y.F.); dengxiaofantju@163.com (X.D.); 3 Tianjin Key Laboratory of High Performance Manufacturing Technology & Equipment, Tianjin 300222, China; 4 School of Mechanical Engineering, Tianjin University, Tianjin 300072, China

**Keywords:** flexible hinge, nano-positioning, guiding mechanism, displacement reduction mechanism, strain displacement measurement

## Abstract

In this paper, a low-cost manual micro- and nano-displacement adjustment mechanism is proposed, based on the principle of flexible hinge transmission and micro-displacement scaling. The manual micro- and nano-displacement platform consists of a micrometer input platform, a nano-output platform, a differential head, and a strain displacement sensor. Firstly, a micro-displacement reduction mechanism based on a flexible beam triangular mechanism and a compact asymmetric flexible beam guiding mechanism are proposed, and a theoretical model is established for static mechanical characteristics, such as the displacement reduction multiplier, guiding stiffness, maximum stress, etc., and this is analyzed and verified by finite element simulation. The software and hardware system of the strain displacement sensor is designed and developed, and the calibration experiments of the strain displacement sensor are completed. Finally, the micro-displacement reduction times, resolution, stability, repeat positioning accuracy, load capacity and travel of the manual micro–nano-displacement platform were analyzed and experimented. The results show that when the input range of the micrometer input platform is 0–1 mm, the travel of the nano-output platform is about 0–16 μm; when a differential head with a step resolution of 2 μm is used to input 2 μm micro-displacement, the minimum displacement output of the nano-output platform is about 35.4 nm; the theoretical and simulated values of the reduction multiple of the micro–nano-displacement are 57.29 and 56.69, respectively; the calibration experiment is performed by the self-developed strain sensors, and capacitive displacement sensors measured the reduction multiples of 57.74 and 62.67, respectively, with high consistency; the vibration range of the platform after the displacement adjustment is about ±30 nm, and the load of 0–300 g has less influence on the output characteristics of the platform.

## 1. Introduction

With the development of nanoscience and technology, micro- and nano-displacement tuning technology, as the basic technology of nanotechnology, is facing opportunities and challenges, and there is a wide range of application needs in the fields of precision instruments [1,2], biomedicine [3,4], and precise positioning in nanoscale manufacturing [5,6], so the design of micro- and nano-positioning platforms has become a cross-disciplinary hotspot of research. In recent years, scientists have designed and developed a variety of technological solutions in key technological fields such as motor drive technology [7,8], micro-displacement guidance technology [9,10] and micro–nano-displacement sensing technology [11,12], and their technological products have gained wide application in different fields by virtue of the advantages of stroke, precision and speed. However, some fields are more sensitive to the size and price of the micro–nano-displacement adjustment system, so it is necessary to design and develop a low-cost, small size micro–nano-displacement adjustment technology.

Precision linear motors, voice coil motors, and piezoelectric ceramic actuators can be used as actuators to realize the adjustment of micro- and nano-displacements [13,14]. Electromagnetic drive motors relying on the feedback control of precision sensors can realize the micrometer displacement control, but due to the friction of the mechanical assembly system, the gap and other nonlinear factors, it is difficult to reach the nanometer level of control accuracy. Piezoceramic actuators, based on the inverse piezoelectric effect under the driving voltage, can obtain nanometer-level motion resolution [15]. In summary, micro/nano-displacement adjustment systems employing electromagnetic actuators impose stringent performance requirements on the actuator components and necessitate dedicated driving electronics; consequently, the total cost for a single-axis configuration typically exceeds several thousand US dollars.

A precision rolling guide and precision air-bearing guide can provide guidance for micro–nanometer displacement by virtue of a smaller friction [16,17], but they need to be installed with nanometer precision displacement sensors, and are usually applied in the field of a larger stroke. The flexible mechanism relies on elastic deformation to achieve micro- and nano-scale displacement adjustments, and as a flexible guiding mechanism without assembly, lubrication, clearance and friction, combined with the driving effect of the piezoelectric ceramic actuator, it is widely used in micro- and nano-displacement platforms [18]. In the piezoelectric-driven micro-displacement system, in order to overcome the insufficiency of the limited stroke of piezoelectric ceramics, most of the studies focus on how to realize the increase in the stroke through the innovative design of the flexible mechanism and the amplification of the mechanical structure, and the commonly used flexible amplification mechanisms include the lever amplification mechanism, the Scott-Russell amplification mechanism, and the bridge-type amplification mechanism, etc. [19]. In contrast to precision-guiding assemblies that entail substantial expenditures in machining, assembly, and maintenance, monolithic flexure mechanisms—fabricated integrally as a single component, requiring no assembly, and operating frictionlessly—can constrain manufacturing costs to within several hundred US dollars while simultaneously achieving an extended travel range through an ingenious mechanism design.

In ultra-precision motion control systems, high-precision displacement sensors are indispensable as displacement measurement and feedback devices. Sensors that can realize micro- and nanometer displacement measurement include laser interferometers, precision scales, capacitive displacement sensors, eddy current displacement sensors and strain displacement sensors, which are applied in different motion systems according to different factors such as their resolution, travel, size and price [20]. Laser interferometers can realize nanoscale displacement measurements but are large and expensive: at least tens of thousands of US dollars [21]. Precision scales can be easily integrated into mechanical systems and are usually used in large travel positioning systems [22]; if the cost is maintained within several thousand US dollars, capacitive displacement sensors and eddy current displacement sensors realize nanoscale displacement measurements in the micrometer range, and are small in size. They are widely used in micro- and nano-displacement platforms [23]; however, the physical dimensions of the associated signal conditioning circuit remain considerable. Strain displacement sensors are widely used in micro- and nano-displacement platforms by measuring the strain of elastic parts that can obtain micro-deformation, which can be used as the measurement and feedback device of flexible hinge-based displacement platforms with a small size and low cost [24]; its signal conditioning circuit can be readily implemented on a compact printed circuit board (PCB) occupying an area of merely a few square centimeters, with a total fabrication cost of less than US $20.

The micro- and nano-displacement adjustment system described above is an automated and precise displacement adjustment technology solution, and the integration of the mechanical system, the drive system and the sensor system makes the overall size larger and more costly, making it difficult to meet the needs of some field occasions for low-cost and small-sized micro and nano-displacement adjustment devices. Manually driven micro-displacement devices are usually only mechanical systems, with a smaller size and lower cost, but assembly accuracy, clearance, friction and other issues lead to poor precision, such as the use of differential screw drive technology, the minimum resolution in the micron level, and difficulty in reaching the nanometer level. In academia and engineering industry, there are few studies and reports of such technology and products.

In this paper, for the demand of low-cost and small-volume micro–nano-displacement adjustment technology, a manual micro–nano-displacement adjustment platform is designed, and a strain displacement sensor system with a digital display function is developed. The full text is arranged as follows: firstly, the design method of the displacement scaling mechanism and guiding mechanism in the manual micro–nano-displacement platform is proposed, and then the design and calibration method of the strain displacement sensor, as well as the composition of the experimental system, are introduced; finally, the performance indexes such as the scaling down multiplier, resolution, stability, repetitive positioning accuracy, and the load characteristic of the micro–nano-displacement platform are experimented and analyzed.

## 2. Design and Analysis of Manual Micro–Nano-Displacement Stage

### 2.1. Design and Analysis of Micro–Nano-Displacement Scaling Structures

**Figure 1:**
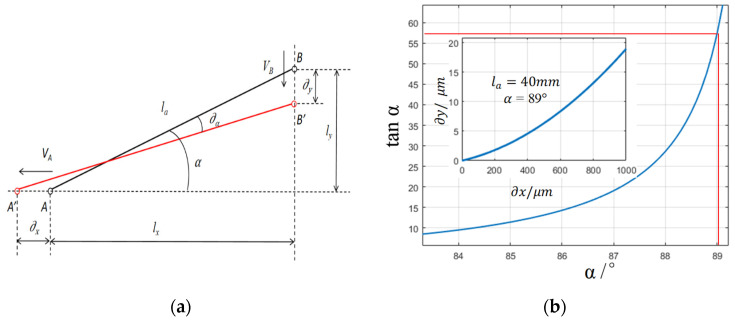
Kinematic analysis of micro-displacement scaling mechanism. (**a**) Kinematic analysis. (**b**) Narrowing scope.

The manually stabilizable input displacements are usually at the micrometer level, such as the differential screw drive that can provide a minimum step displacement of 2 μm. In order to realize the output of nanoscale step displacements, a micro-displacement scaling structure needs to be designed. In this paper, a displacement scaling mechanism based on a flexible hinged triangular mechanism is proposed, as shown in Figure 1a. The displacement scaling mechanism consists of a long diagonal flexible beam: one end, the input platform, only has X-direction degrees of freedom; the other end, the output platform, only has Y-direction degrees of freedom; and the flexible beam is the tensile to drive the output end to generate the displacement when the input displacement ∂x is acted upon by the displacement ∂y, as shown in the figure, it has shifted from the initial position of the blue line to that of the red line. From the geometric relationship, it can be seen that when generating micro-displacement movement, the relationship between ∂x, ∂y and the angle of the flexible beam α is as follows:

(1) $$\tau =\frac{\partial x}{\partial y}=\frac{V_{A}}{V_{B}}=\frac{\omega l_{x}}{\omega l_{y}}=\frac{l_{x}}{l_{y}}=\tan\alpha$$

It can be seen from the formula that when the angle α is close to 90°, a larger bit reduction ratio can be obtained under the condition of limited size of the overall mechanism, as shown in Figure 1b. Specifically, with *l_a_* = 40 mm and α = 89°, an input displacement in the X-direction ranging from 0 to 1000 μm produces an output displacement of 0 to 19 μm, yielding a reduction ratio of approximately 55. Based on the kinematic analysis of the structure illustrated in Figure 1a, a mathematical model (2) correlating the input and output displacements is derived, and the corresponding relationship curve is presented in Figure 1b.

(2) $$\partial y=l_{a}\sin\alpha -\sqrt{{l_{a}}^{2}-{\left(l_{a}\cos\alpha +\partial x\right)}^{2}}$$

**Figure 2:**
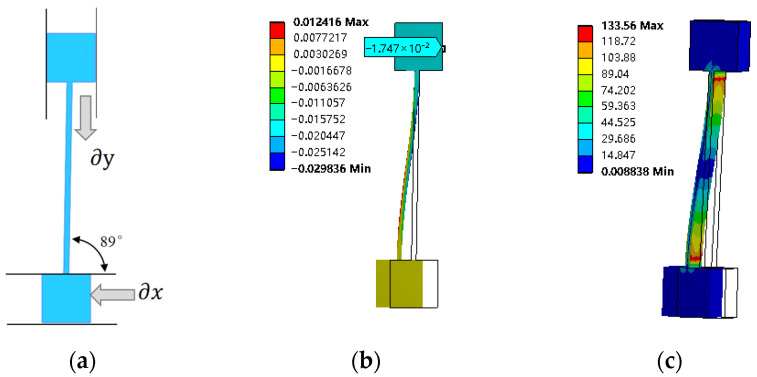
Simulation analysis of micro-displacement scaling mechanism. (**a**) Micro-displacement scaling structures. (**b**) Micro-displacement scaling simulation. (**c**) Micro-displacement scaling stress simulation.

The flexible beam motion transformation mechanism is established by Solidworks 2024 software, as shown in Figure 2a, and the length, width and height of the inclined beam are 40 mm, 1 mm and 10 mm, respectively, and the initial angle α is 89°. A 1 mm input displacement is set in the direction of ∂x at the lower input end, and the micro-displacement scaling distributions and the stress distributions are obtained through the finite element simulation, as shown in Figure 2b,c. The simulated output displacement ∂y of the output end is 17.38 μm, and the scaling factor is 57.24, which is slightly smaller than the theoretical value of 57.29, because the angle of the inclined beam has been made smaller when the 1 mm displacement is input into the din direction, and thus the simulated scaling factor is obtained by inputting 1 mm in the din direction, which is slightly smaller. From the stress distribution cloud (Figure 2c), it can be seen that the stresses at the two ends of the inclined beam are the largest, and there is no stress in the middle.

### 2.2. Design and Analysis of Micro–Nano-Displacement Guide Mechanism

#### 2.2.1. Design of Output Platform Guide Mechanism and Analysis

**Figure 3:**
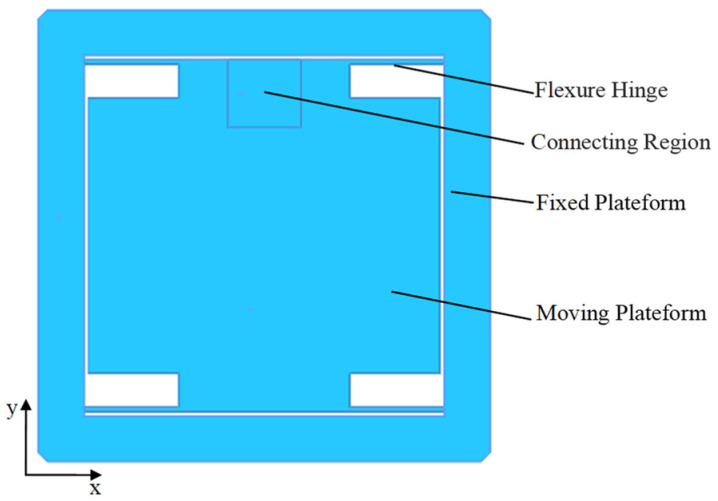
Output platform and guide mechanism.

From the above analysis, it can be seen that the output platform with nanoscale resolution has only degrees of freedom in the direction of motion and the travel is at the micron level, so the guiding mechanism for double-sided pair molding can be designed based on the principle of flexible hinge transmission, as shown in Figure 3. When the connection region in Figure 3 is joined to the output platform depicted in Figure 2a, the moving platform, guided by four flexure beams, can achieve translational motion along the *Y*-axis at the micro/nanometer scale. The length *l*, thickness *t* and height *h* of the four flexible hinges are 15 mm, 1 mm and 8 mm, respectively. Since the platform can be regarded as a rigid body, only the stiffness of the four right-angle flexible hinges is calculated in the modeling process. Since the flexible hinges can be equivalent to a spring in tension, the stiffness of the whole motion guiding mechanism is the parallel tensile stiffness of the four equivalent springs.

The stiffness of the output platform guide mechanism is as follows:

(3) $$k_{g}=4k=\frac{4Eht^{3}}{L^{3}}$$

where *L* denotes the length of the flexible hinge, *h* is the width of the flexible hinge, and *t* is the thickness of the flexible hinge.

#### 2.2.2. Input Platform Guide Mechanism Design and Analysis

**Figure 4:**
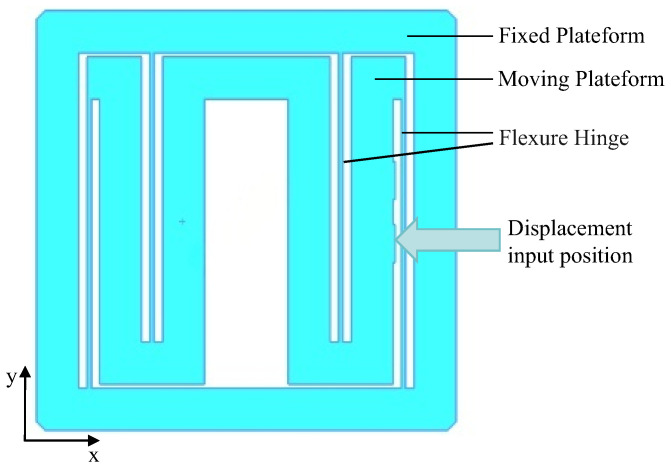
Input platform and its guiding mechanism.

According to the requirements of the motion transformation mechanism illustrated in Figure 1, the overall structure’s input platform is connected to the input platform shown in Figure 1a. This configuration enables translational motion within a millimeter range along the *X*-axis while necessitating a compact form factor. In order to achieve millimeter-level travel motion in a limited space, a bilateral asymmetric flexible beam guiding mechanism is designed, as shown in Figure 4. The input platform consists of a static platform, a movable platform and a flexible hinge: the movable platform is W-shaped inside the static platform, the displacement input position is located in the middle of the movable platform, and the middle cavity is designed to install the above-mentioned micro-displacement transforming flexible beams. Four flexible beams are connected to the static and movable platforms in the left and right groups to ensure that the movable platform has only one translational degree of freedom.

**Figure 5:**
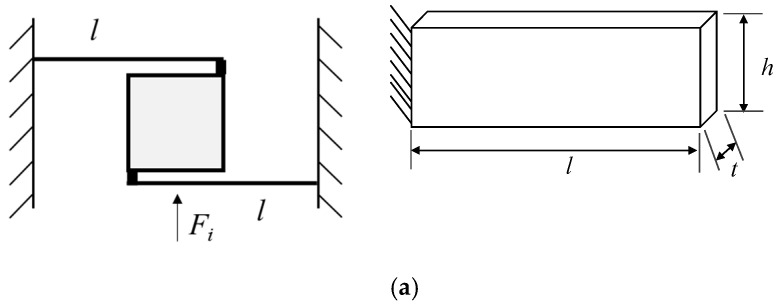

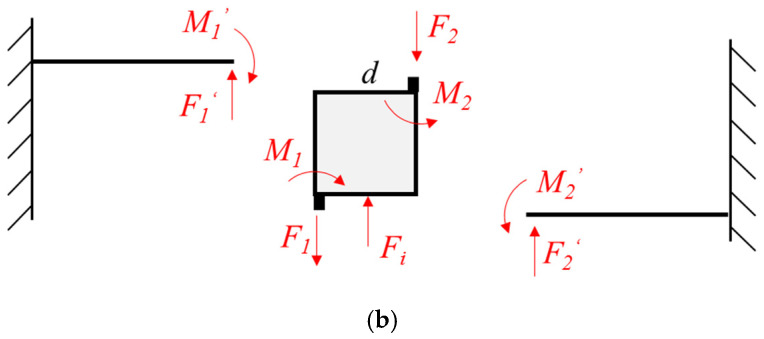
Bilateral asymmetric flexible beam positioning platform sketch and force analysis. (**a**) Sketch of an asymmetric flexible beam positioning platform. (**b**) Force analysis.

The asymmetric guiding mechanism based on flexible beams is taken as the research object, as shown in Figure 5a,b, to analyze the relationship between the system stiffness, travel and key dimensions. The length, thickness and height of the flexible thin plate beam are *l*, *t* and *h*, and the driving force is applied at the middle position of the moving platform to analyze the force on the system and establish the equilibrium equations.

(4) $$\left\{\begin{array}{l} F_{i}=F_{1}+F_{2}\\ F_{1}\frac{d}{2}-M_{1}=F_{2}\frac{d}{2}-M_{2}\\ F_{1}=F_{2}=\frac{F_{i}}{2} \end{array}\right.$$

Because it is a symmetrical structure, the moving platform only has translational motion in the driving direction, and the beam end angle of rotation is zero, i.e., $\theta_{F1}=\theta_{M1}$, which can be obtained as follows:

(5) $$\left\{\begin{array}{c} \frac{F_{i}l^{2}}{2EI}=\frac{M_{1}l}{2EI}\\ M_{1}=M_{2}=\frac{F_{i}l}{4} \end{array}\right.$$

The end deflection of a flexible thin plate beam connected to a moving platform w is as follows:

(6) $$w=\frac{M_{1}l^{2}}{2E_{I}}=\frac{F_{1}l^{3}}{3E_{I}}=\frac{F_{1}l^{3}}{2Et^{3}}$$

where *E* is the Young’s modulus of the flexible hinge material, $I=(ht^{3})/12$ is the moment of inertia of the cross-section of the thin plate beam.

Thus, the stiffness of a pair of asymmetric flexible beams, *k*, can be obtained as

(7) $$k=\frac{F_{i}}{w}=\frac{2Eht^{3}}{l^{3}}$$

To ensure the performance of the flexible beam, the internal stress of the flexible beam within the travel range should be less than the allowable stress of the material:

(8) $$\sigma_{max}=\frac{M_{1}t}{2I}=\frac{3Etw}{l^{3}}\leq \left[\sigma \right]$$

To increase the stiffness, n pairs of asymmetric flexible beams can be connected in parallel with the stiffness:

(9) $$k_{n}=\frac{2nEht^{3}}{l^{3}}$$

The input force is:

(10) $$F_{i}=k_{n}w=\frac{2nEht^{3}w}{l^{3}}$$

**Figure 6:**
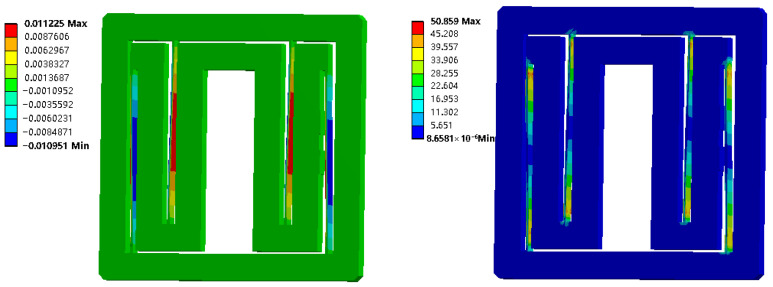
Simulantion analysis of input platform and its guiding mechanism.

Assuming that the length *l*, thickness *t* and height *h* of the four flexible beams are 65 mm, 1 mm and 8 mm, respectively, *n* is 2, and the material is set as aluminum alloy 7075. According to the theoretical model presented above, the input stiffness is determined to be 8.155 N/mm, indicating that an actuation force of 8.155 N is required to achieve a 1 mm input displacement. The static simulation is carried out by ANSYS15.0. When 1 mm displacement in the X-direction is input to the moving platform, the finite element simulation results are shown in Figure 6, and the coupling displacement of the moving platform in the Y-direction is zero, which proves a single translational degree of freedom of the moving platform. The stress distribution of the flexible beams conforms to the distribution characteristics of the guide beam, and its maximum stress value is 50.859 MPa, which meets the use requirements. The reaction force measured at an applied input displacement of 1 mm is 7.081 N, yielding an experimental input stiffness of 7.081 N/mm, which is in close agreement with the theoretical value of 8.155 N/mm.

### 2.3. Integration of Micro–Nano-Displacement Scaling Mechanism with Guidance Mechanism

**Figure 7:**
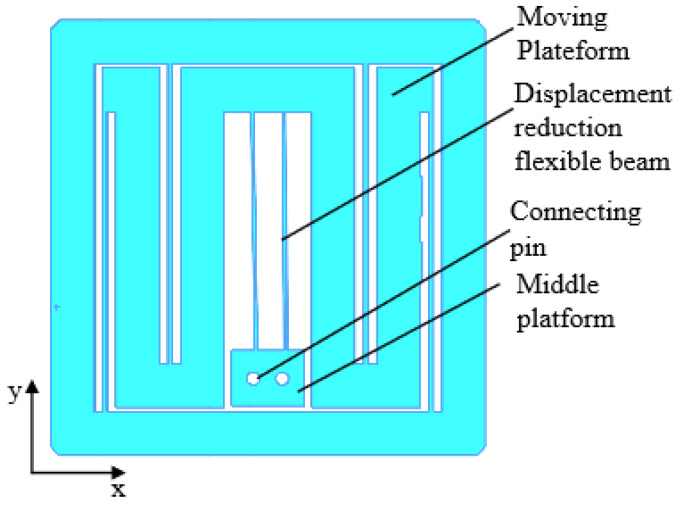
Integration of micro–nano-displacement scaling mechanism with guide mechanism.

In order to realize the compact design of the overall structure, the micron displacement scaling mechanism is integrated in the middle of the guiding mechanism of the input platform, as shown in Figure 7. Two flexible beams are used as the displacement scaling mechanism to connect the moving platform of the input platform and the intermediate platform, and the intermediate platform is connected to the moving platform of the nano-output platform through the interference connection of the pins; as a result, the moving platform of the micrometer input platform has only the degree of freedom in the X-direction, and the intermediate platform, like the nano-output platform, has only the translational degree of freedom in the Y-direction. During the motion, the moving platform is subject to the tension of the intermediate platform in the Y-direction, and the stiffness of the moving platform in the Y-direction is extremely large, so the translational interference of the intermediate platform on the moving platform is negligible.

### 2.4. Manual Micro–Nano-Displacement Stage Design

**Figure 8:**
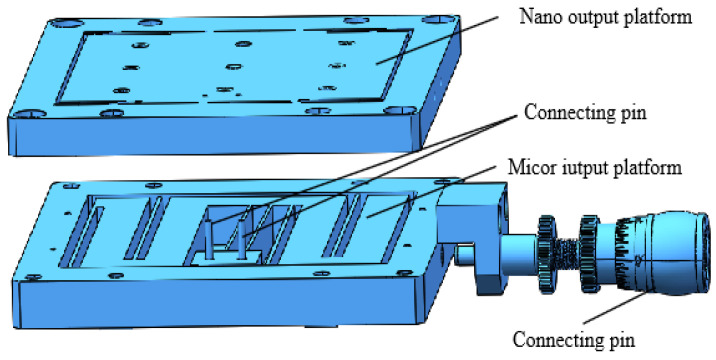
Composition of manual micro–nano-displacement stage.

The structure of the manual micro–nano-displacement platform is shown in Figure 8, which consists of a nano-output platform, a micrometer input platform, a micro–nano-displacement transformation mechanism, a microtome, a microtome holder, a bottom cover plate and a connecting pin. The micro–nano-displacement transformation mechanism adopts two parallel long straight flexible beams with an angle of 89°, which are embedded in the micrometer input platform as an integrated structural design; the connection between the nano-output platform and the micro–nano-displacement transformation mechanism is realized through the interference of the pins, and the micrometer input platform is provided with the micrometer input by the micrometer head. The outer frame of the micrometer input platform and the outer frame of the nano-output platform are connected by bolts, and the overall size is 130 mm × 100 mm × 18 mm.

**Figure 9:**
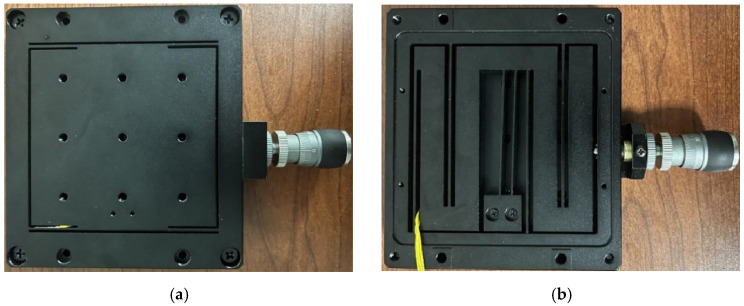
Physical drawing of the manual micro–nano-positioning platform. (**a**) Nano-output platform. (**b**) Micron input platform.

Aluminum alloy 7075 is selected as the platform material and processed by the CNC and wire-cutting process; the assembled prototype is shown in Figure 9. Figure 9a is the front side of the prototype and Figure 9b is the back side of the prototype.

### 2.5. Finite Element Analysis

**Figure 10:**
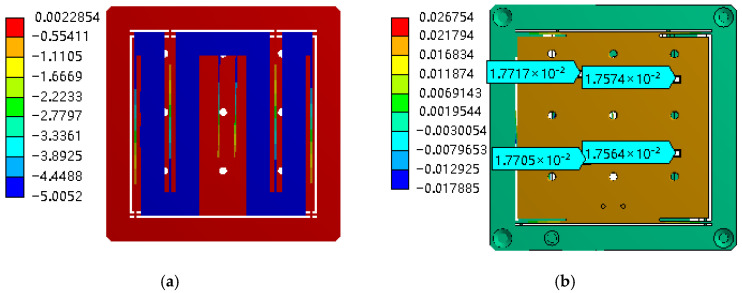
Output displacement distribution of the micro–nano platform at 5 mm input displacement. (**a**) Distribution of deformation at 1 mm input displacement of the nano-output stage. (**b**) Output displacement distribution of the nanoplatform at 1 mm input displacement.

The finite element analysis of the manual micro–nano-displacement platform is mainly performed by ANSYS 15.0 Workbench 15 for the deformation and stress analysis of the flexible beam. The material of the displacement platform is selected as aluminum alloy 7075, and 1 mm displacement is applied in the X-direction of the input platform to simulate the input amount of the micrometer head. As shown in Figure 10a, the overall displacement of the rigid structure in the middle of the input platform is 1 mm, and the asymmetric flexible beams achieve the guiding effect. Figure 10b shows the output displacement distribution of the nano-stage at 1 mm input displacement, and the displacement of the output stage in the Y-direction is 17.56~17.72 μm, from which the displacement reduction multiplier is about 56.43~56.95, counted as the average of 56.69.

**Figure 11:**
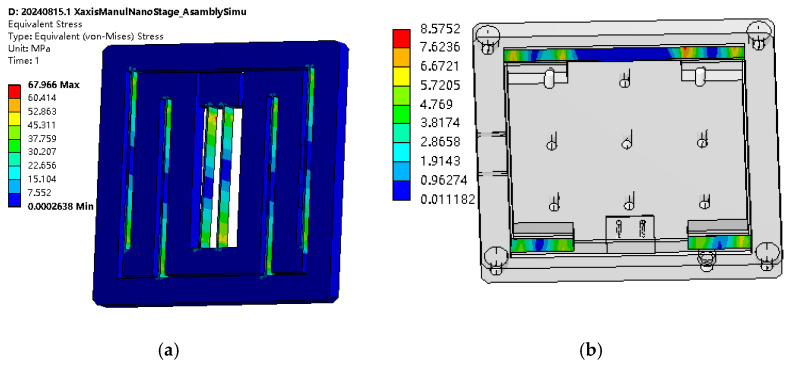
Stress simulation at 1 mm input displacement. (**a**) Input platform stress distribution. (**b**) Output platform stress distribution.

Figure 11 shows the stress distribution of the input platform and output platform at 1 mm input displacement, respectively. It can be seen that the maximum stress of 67.966 MPa occurs in the intermediate diagonal beam, which is much smaller than the permissible stress of the material, and the maximum stress of the guiding flexible hinge of the output platform occurs in the vicinity of the root of the straight beam, where the strain gauges can be affixed as the displacement sensors to achieve the precision adjustment and real-time display of the displacement. Based on the simulation results, the required driving force is 9.762 N, with the excess component dedicated to overcoming the reaction force exerted by the Y-direction guiding flexure hinges of the output platform. This driving force remains well within the 20 N load capacity specification of the adopted differential micrometer head.

From the above simulation results, it can be seen that the input range of this manual nano-displacement platform is 0~1 mm, and the output range of the nano-output platform is 0~18 μm, and if a differential micrometer head with a step resolution of 2 μm is used for the drive, a minimum displacement output of 40 nm can be achieved.

## 3. Design of Experimental System for Micro–Nanometer Displacement Measurement

### 3.1. Strain Measurement System Design

**Figure 12:**
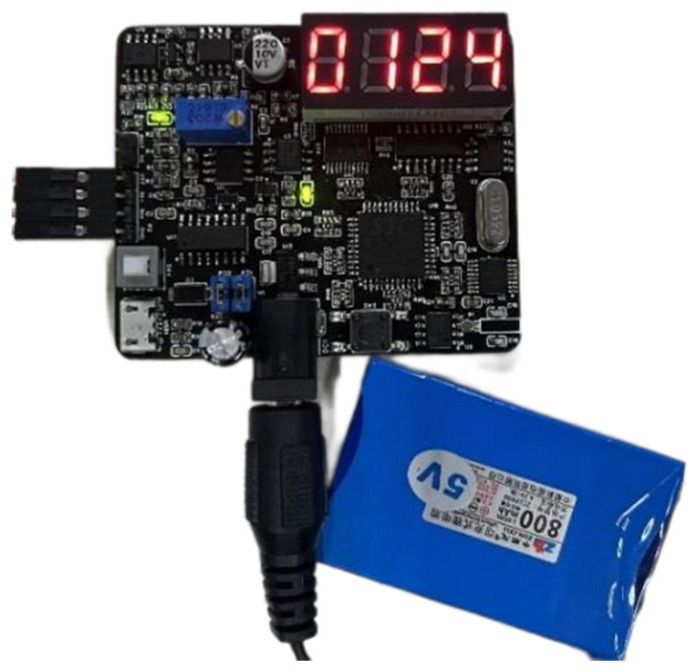
Strain measurement system composition and physical diagram.

The manual nano-positioning platform designed in this paper is a low-cost motion control device, and a micro-displacement measurement system based on strain measurement is developed to control the cost and achieve precise and accurate micro-displacement adjustment. The manual nano-positioning stage realizes the transmission and guidance of micro-displacement through the elastic deformation of the flexible beam, which is proportional to the size micro-displacement, so micro-displacement measurement can be realized by detecting the strain. Strain detection is a means of deformation detection with a certain degree of accuracy and a low cost, which can be used as a micro-displacement sensor. The structure and physical object of the strain measurement system is shown in Figure 12, with the digital display module, AD/DA communication module, program storage and other modules and mobile power supply, and the digital display module can display the adjusted micro–nano-displacement in real time.

**Figure 13:**
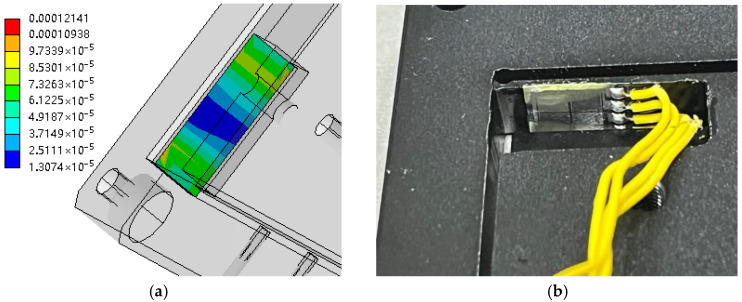
Finite element analysis of the strain of the flexible beam of the output platform and the position of the strain gauges to be attached. (**a**) Strain analysis. (**b**) Actual position of the strain gauges to be attached.

Determining where to affix the strain gauges is an important issue when strain measurements are used to monitor nanoscale displacements of the output platform. As mentioned before, the output platform uses four symmetrically distributed flexible beams to create translational guidance, so any one of the flexible beams can be used as the detection structure, and according to the knowledge of material mechanics, in the flexible beams with the role of translational guidance, the maximum strain occurs at the ends of the beams, and the middle strain is zero. During the finite element simulation, when 1 mm displacement is fed to the input platform, the strain distribution of the guided flexible beam of the output platform is shown in Figure 13a, and the maximum strain occurs at the roots of both sides of the beam (0.012%). In order to improve the sensitivity of the micro–nano-displacement detection, the strain gage pasting position should be selected at the maximum strain, and the actual pasting position is shown in Figure 13b.

**Figure 14:**
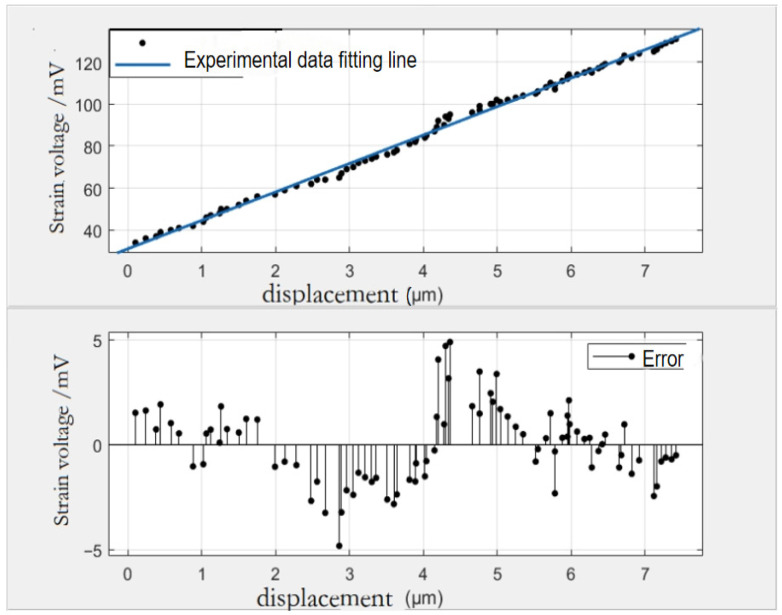
Displacement vs. strain voltage fit, residual plots.

Calibration experiments were conducted on the developed strain sensor measurement system to establish the relationship between the strain and the strain voltage signal of the strain displacement sensor. Strain gauges of the same type are pasted onto the flexible hinge of the self-developed piezoelectric-driven nano-displacement platform, and at the same time, the displacement signals are measured by the capacitive displacement sensors with the self-developed strain measurement sensors, from which the relationship between the displacement signals detected by the capacitive displacement sensors and the strain voltage signals of the self-developed strain displacement sensors can be established, as shown in Figure 14. Fitting the displacement and strain voltage quantities, the displacement–strain voltage relationship equation is established: F(X) = P1x + P2, where P1 is 13.53, P2 is 31.13, and the root mean square error RMSE is 1.856, and R2 is 0.9957, which is a good fit. This relational equation is written into the microcontroller program of the detection system and the detected displacement values are directly displayed in the display module.

### 3.2. Experimental System Construction

In order to test the validity of the micro-displacement adjustment of the manual micro–nano-displacement platform and to calibrate the above strain measurement system, this paper adopts high-performance capacitive displacement sensors (Type) and laser displacement sensors (Keens IL-S065), and builds an experimental system of micro–nano-displacement testing machine calibration.

**Figure 15:**
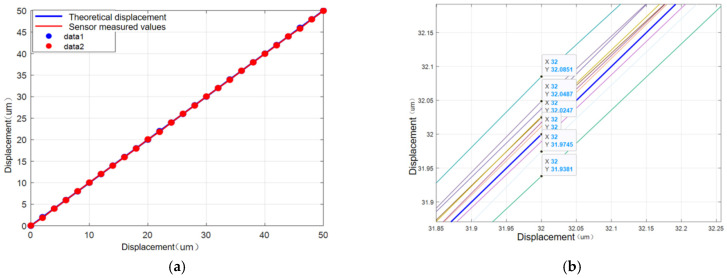
Micrometer head theoretical and measured values. (**a**) Micrometer head theoretical and measured values. (**b**) Theoretical and measured values of multiple micrometer heads.

The micrometer head (Type) is used for micro-displacement input, and a large-stroke laser displacement sensor is used for input calibration testing of the micrometer head input. The average relative error of the measured values of the 10 sets of data is about 0.14%, and the micrometer head can be stably input to micro-displacements with a resolution of 2 μm within the range of the elastic loading of the drive micrometer stage, and the test results are shown in Figure 15.

**Figure 16:**
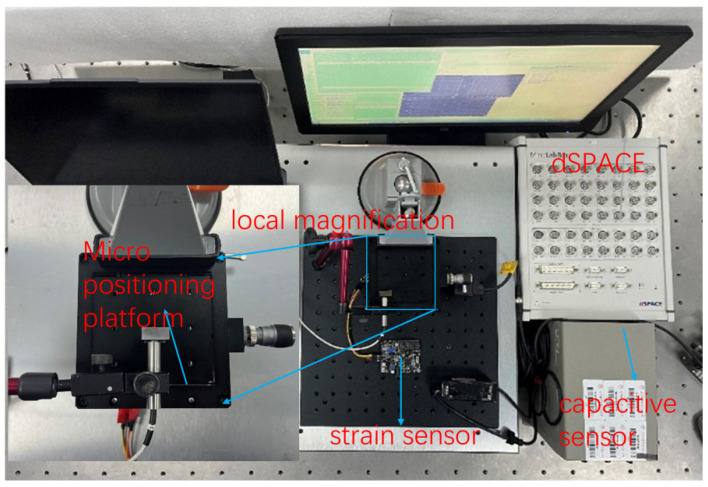
Measurement experiment of micro–nano-positioning platform.

For the manual micro–nano-displacement platform designed in this paper, the micro-displacement input is realized by the differential head, while the output displacement is detected by the capacitive displacement sensor and strain displacement sensor, and the experimental system is shown in Figure 16.

## 4. Performance Analysis of Manual Micro–Nano-Displacement Platforms

### 4.1. Micro-Displacement Input/Output Experiments

**Figure 17:**
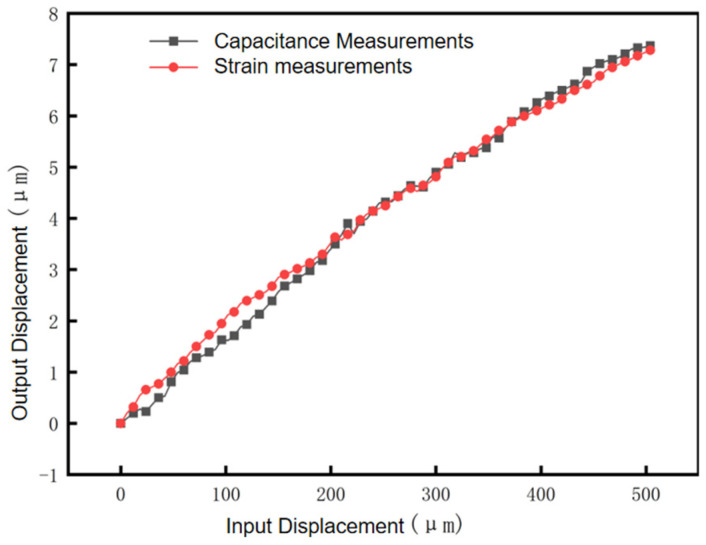
The 6 μm step displacement capacitance and strain measurements.

During the experiment, the micrometer head provides a total input travel of 0–504 μm in 6 μm step displacement, and the computer records the Lion Precision capacitive displacement sensor (Range ±25 μm, resolution 0.2 nm) data and strain sensor (Range ±1000 με, resolution 0.5 με) data through the dSPACE data acquisition system. The dSPACE sampling frequency is 10 kHz, the signal filtering is a fourth-order Butterworth low-pass filter at 500 Hz, the experimental environment is a temperature-controlled laboratory (20 ± 0.5 °C), and the vibration isolation is an active pneumatic isolation table with <50 nm floor vibration. The experimental results are shown in Figure 17, in which the black line segment and the red line segment are the output displacements collected by the capacitive displacement sensor and the strain displacement sensor, respectively, and the consistency of the two data lines is very high, which indicates that the accuracy of the developed strain displacement sensor is higher; the reduction in the micro–nanometer displacement calculated from the experimental data is about 57.74~62.67, which is in agreement with the simulation value of 56.43~56.95 and the theoretical value of 57.29.

### 4.2. Resolution

**Figure 18:**
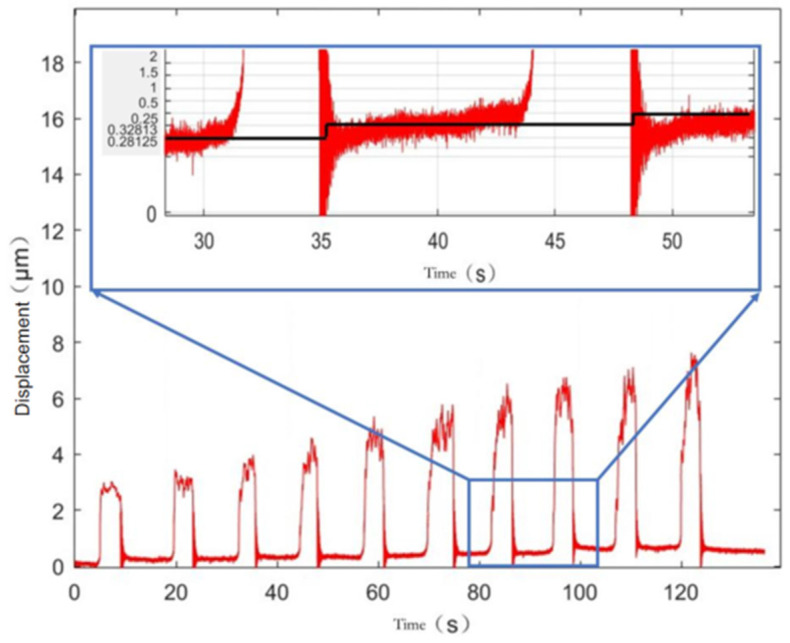
Overall motion resolution of the micro–nano-positioning platform.

Motion resolution is a very important performance metric for a micro–nano-displacement stage. The micro–nano-displacement stage is driven by the differential head to input the smallest 2 μm step displacement, and the step displacement output is measured by the capacitive displacement sensor, i.e., the minimum motion resolution of the micro–nano-positioning stage. The measurement results are shown in Figure 18, and the motion resolution of the micro–nano-positioning platform is basically maintained at 35.3 nm, i.e., the micro–nano-displacement reduction is about 56.66 times, which is basically consistent with the previous experimental results.

### 4.3. Stability

As can be seen in Figure 18, when the differential head input is manually adjusted in steps of 2 μm each time, the capacitive displacement sensor detects a sharp (2–7 μm) vibration with large transient overshooting of the nano-output platform, but the nano-output platform is relatively stable at the end of the adjustment.

**Figure 19:**
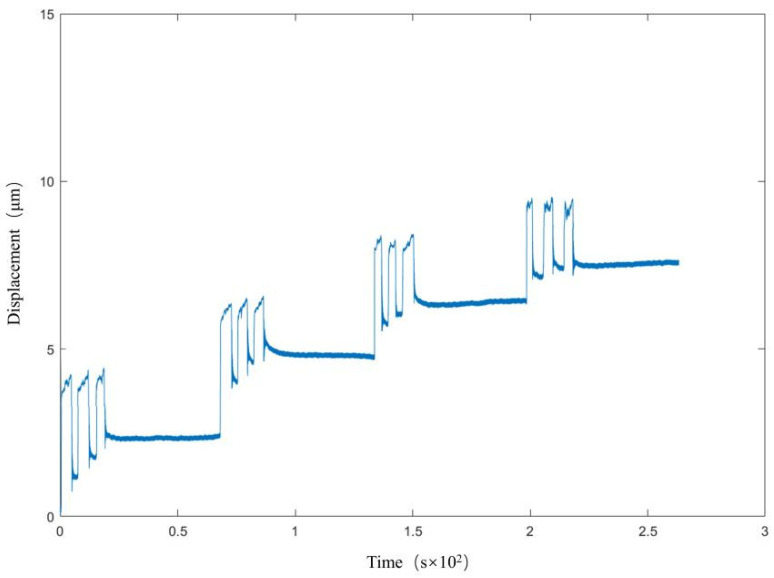
Stability of the micro-localization platform.

In order to test the stability of the platform, the differential head was adjusted to be stationary at the positions of 120 μm, 240 μm, 360 μm and 480 μm of input displacement for one minute, and the displacement change in the nano-output platform was measured by the capacitive displacement sensor. Figure 19 shows the output displacements at four different input displacements, and the nano-output platform can remain stable at its location for one minute, indicating that the micro–nano-positioning platform has good stability, with a vibration range of about ±30 nm.

### 4.4. Repeat Positioning Accuracy

**Figure 20:**
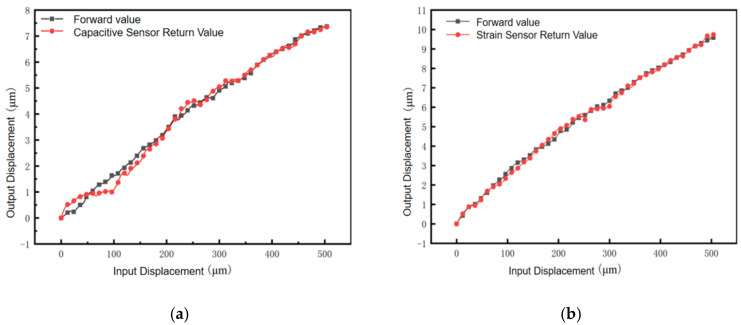
Forward and reverse position relationships. (**a**) Capacitive sensor forward and return curves. (**b**) Strain transducer forward and return curves.

Capacitive displacement sensors and strain displacement sensors were used to test the repetitive positioning accuracy of the micro–nano-displacement stage, respectively, and the micrometer head reciprocated within the range of 0~504 μm with a stepping distance of 6 μm. The results are shown in Figure 20: the black square curve is the measurement curve when stepping in the forward direction, and the red circle curve is the measurement curve when the capacitive sensor and the strain sensor reduce the stepping in the opposite direction. The experimental data show that the hysteresis error C1 of the capacitive sensor reciprocation is 0.126% in the 0–504 μm reciprocation measurement data. The accuracy of the capacitance sensor in the figure is too high and is susceptible to interference from the external environment, resulting in large ups and downs in the measurement data. The corresponding hysteresis error C2 of the strain sensor reciprocation is 0.11%.

### 4.5. Repeatability

**Figure 21:**
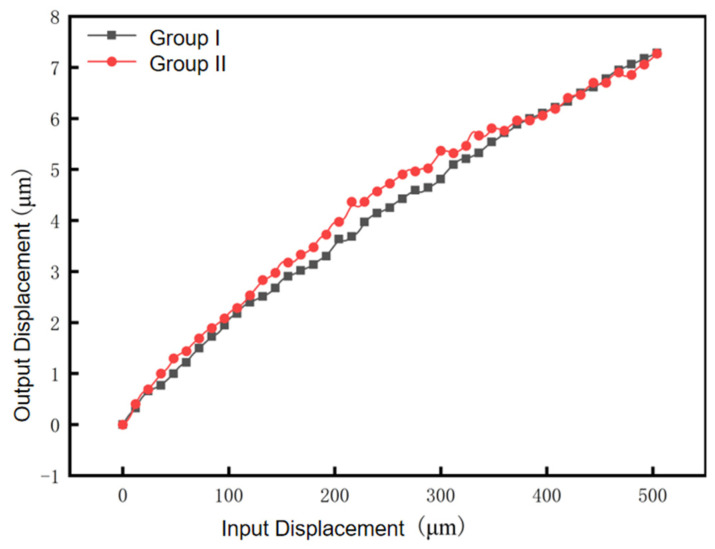
Strain transducer repeatability.

The repeatability of the strain sensor refers to the degree of stability and the consistency of the output value when the sensor measures the input within the same range several times under the same operating conditions, reflecting the random error of the sensor when analyzing the repeatability of the sensor’s needs to calculate the maximum difference between the mutual offset of the output of the same input in the same direction of travel and the ratio of the total input travel. This is shown in Figure 21. We calculate the repeatability error F of the strain sensor as 0.6813/504 × 100% = 0.14%.

### 4.6. Load Experiments on Micro–Nano-Positioning Platform

**Figure 22:**
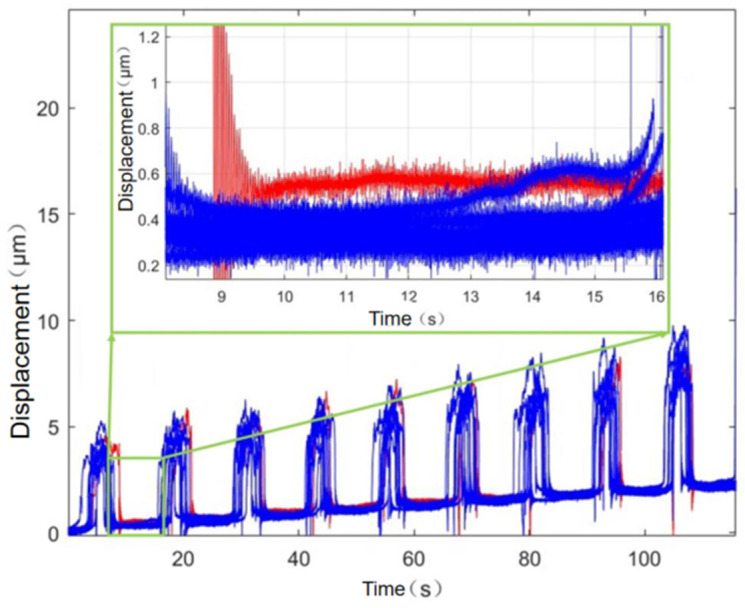
Comparison of load experiment data curves.

The nano-output platform is supported by four flexible beams, and when the nano-output platform is loaded by the load, the elastic deformation of the four flexures is likely to receive the effect of the load. In order to investigate the loading performance of the nano-positioning platform, a standard gram-valued weight is used as a loading tool to experiment on its load resistance. In the experiments, a stabilized 10 μm step input was provided by a differential head and the corresponding displacement output was detected by a capacitive displacement sensor. The output displacements under no load, 100 g, 200 g, and 300 g loads are tested, respectively. Eleven sets of measurement data are shown in Figure 22, where the red curve is the displacement curve of the micro–nano-positioning platform under no load, the blue curve indicates the displacement curve under multiple loads, and the red and blue curves overlap many times and have no obvious displacement deviation. It can be clearly seen that the micro–nano-positioning platform can be used in the case of no load and multiple loads. It can be clearly seen that the micro–nano-positioning stage shows good load resistance under no load and multiple loads, and the average difference between the 10 sets of inputs with loads and without loads is calculated to be 0.23 μm.

### 4.7. Itinerary

From the simulation results, it can be seen that when the input range of the manual nano-displacement platform is 0~1 mm, the output range of the nano-output platform is 0~18 μm, and the maximum stress of 101.95 MPa is much smaller than the permissible stress 310 MPa, so the input range can be set to within 1 mm.

From the above experimental results, it can be seen that the input range is 0~7.36 μm at the input range of 0~504 μm, which is larger than the minimum adjustment of 2 μm for the differential head, so the manual micro–nanometer displacement stage designed in this paper can realize the 50 nanometer level displacement adjustment in the range of 0~7 μm.

For the strain displacement sensor developed in this paper through the previous calibration experiments, to find out the amount of strain voltage corresponding to the measured displacement in the experiment, we analyze that with every increase of 0.088 μm, it increases the amount of voltage by 1.169 mV; the sensor can be increased to 3600 mv, and the for the calculation of strain sensors, we have to measure the stroke for 0–271 μm.

## 5. Conclusions

In this paper, a low-cost differential head-driven nanoscale displacement adjustment platform is designed and developed based on the micro-displacement scaling principle of the flexible hinge triangular mechanism, which mainly proposes a micro–nano-displacement reduction mechanism with a larger multiplier, as well as an asymmetric flexible hinge drive mechanism with a millimeter level, and a strain displacement sensor with digital display function is designed and developed based on the strain-sensing principle. Through theoretical calculation, simulation analysis and experimental verification, the designed manual micro–nano-displacement platform can transform the manual input displacement of 2 μm into the output displacement of 35 nm, and can help us to achieve the micro-displacement adjustment and positioning of 35 nm within the range of 0–7 μm; the stability experiments show that the platform only vibrates ± 30 nm after the completion of the drive feed, and no creep, rebound or other phenomena are found at 1 min; load experiments show that a 300 g load does not have a significant effect on the stability of the platform and positioning the platform; with the digital display function, the strain displacement sensor can effectively measure the position of the feedback output platform, so the micro–nanometer displacement adjustment provides a low-cost, effective and reliable solution.

## Data Availability

The original contributions presented in this study are included in the article. Further inquiries can be directed to the corresponding author.
